# What is conservation physiology? Perspectives on an increasingly integrated and essential science^†^

**DOI:** 10.1093/conphys/cot001

**Published:** 2013-03-13

**Authors:** Steven J. Cooke, Lawren Sack, Craig E. Franklin, Anthony P. Farrell, John Beardall, Martin Wikelski, Steven L. Chown

**Affiliations:** 1 Fish Ecology and Conservation Physiology Laboratory, Department of Biology and Institute of Environmental Science, Carleton University, 1125 Colonel By Drive, Ottawa, ON, Canada K1S 5B6; 2 Department of Ecology and Evolution, University of California Los Angeles, 621 Charles E. Young Drive South, Los Angeles, CA 90095, USA; 3 School of Biological Sciences, The University of Queensland, Brisbane, Queensland 4072, Australia; 4 Department of Zoology and Faculty of Land and Food Systems, University of British Columbia, 6270 University Boulevard, Vancouver, BC, Canada V6T 1Z4; 5 School of Biological Sciences, Monash University, Victoria 3800, Australia; 6 Max Plank Institute of Ornithology, D-78315 Radolfzell, Germany

**Keywords:** Conservation physiology, conservation science, environment, mechanisms, resource management

## Abstract

Globally, ecosystems and their constituent flora and fauna face the localized and broad-scale influence of human activities. Conservation practitioners and environmental managers struggle to identify and mitigate threats, reverse species declines, restore degraded ecosystems, and manage natural resources sustainably. Scientific research and evidence are increasingly regarded as the foundation for new regulations, conservation actions, and management interventions. Conservation biologists and managers have traditionally focused on the characteristics (e.g. abundance, structure, trends) of populations, species, communities, and ecosystems, and simple indicators of the responses to environmental perturbations and other human activities. However, an understanding of the specific mechanisms underlying conservation problems is becoming increasingly important for decision-making, in part because physiological tools and knowledge are especially useful for developing cause-and-effect relationships, and for identifying the optimal range of habitats and stressor thresholds for different organisms. When physiological knowledge is incorporated into ecological models, it can improve predictions of organism responses to environmental change and provide tools to support management decisions. Without such knowledge, we may be left with simple associations. ‘Conservation physiology’ has been defined previously with a focus on vertebrates, but here we redefine the concept universally, for application to the diversity of taxa from microbes to plants, to animals, and to natural resources. We also consider ‘physiology’ in the broadest possible terms; i.e. how an organism functions, and any associated mechanisms, from development to bioenergetics, to environmental interactions, through to fitness. Moreover, we consider conservation physiology to include a wide range of applications beyond assisting imperiled populations, and include, for example, the eradication of invasive species, refinement of resource management strategies to minimize impacts, and evaluation of restoration plans. This concept of conservation physiology emphasizes the basis, importance, and ecological relevance of physiological diversity at a variety of scales. Real advances in conservation and resource management require integration and inter-disciplinarity. Conservation physiology and its suite of tools and concepts is a key part of the evidence base needed to address pressing environmental challenges.

## Abstract (precis)

The definition of ‘conservation physiology’ is refined to be more inclusive, with an emphasis on characterizing diversity, understanding and predicting responses to environmental change and stressors, and generating solutions. The integrative discipline is focused on mechanisms and uses physiological tools, concepts, and knowledge to advance conservation and resource management.

We define conservation physiology as: ‘An integrative scientific discipline applying physiological concepts, tools, and knowledge to characterizing biological diversity and its ecological implications; understanding and predicting how organisms, populations, and ecosystems respond to environmental change and stressors; and solving conservation problems across the broad range of taxa (i.e. including microbes, plants, and animals). Physiology is considered in the broadest possible terms to include functional and mechanistic responses at all scales, and conservation includes the development and refinement of strategies to rebuild populations, restore ecosystems, inform conservation policy, generate decision-support tools, and manage natural resources.’

## Introduction

The idea that physiological knowledge can inform conservation is not new. Although a search for the phrase ‘conservation physiology’ within Web of Science yields <30 papers, the pages of conservation journals include physiological content, while those of physiological journals include population management and conservation. Indeed, prior to the middle of the 20th century, ecology and physiology had largely been regarded as synonymous (Gaston *et al.*, 2009), resulting in frequent application of physiological approaches to investigations of population dynamics (e.g. Cook, 1924; Payne, 1926; Sacharov, 1930; Andrewartha and Birch, 1954). In many ways, the August Krogh principle for physiological adaptation, which was coined over a century ago (‘for … a large number of problems there will be some animal of choice, or a few such animals, on which it can be most conveniently studied’), is derived from the marvellous match of an animal's physiology with its environment (Hochachka and Somero, 2002). Fry (1947) recognized six categories of effects (i.e. factors) for fish in which the environment and physiology interacted. Much earlier, Shelford (1911) provided a comprehensive treatment of environmental factors shaping plant and animal distributions, with much focus on physiological mechanisms, poorly explored as many of them were at that time.

The discussion of physiology in a conservation context began to increase in the early 1990s, perhaps reflecting the growing appreciation for the significance of the field during and after the meeting organized by Wilcox and Soulé at which the term ‘conservation biology’ was first introduced. Although the journal *Biological Conservation* has existed since the late 1960s, and *Conservation Biology* since 1987, work with explicit reference to physiology in the abstract only began to appear in the 1990s. Likewise, in the fourth edition of his seminal book, Prosser (1991) argued the need for greater physiological information on stressed species given drastic environmental change and population reductions. A year later, in reviewing the iconoclastic 1987 volume by Feder *et al*., Gnaiger (1992) devoted his final section to the need for physiology to be concerned with human impacts. A parallel shift has also occurred in the field of toxicology and the sub-field of environmental toxicology. At one time, these areas were concerned with lethality as a measurement and regulatory end-point. Today, the importance of sub-lethal toxicity is well recognized, and books have been devoted to the mechanistic physiology that underlies toxicity (e.g. for fishes, Wood *et al.*, 2012a, b). The reason for these shifts is simple; there is a much better appreciation of animal and plant physiology, especially in relationship to their environments.

Such recognition of the significance of physiology for conservation has grown in primary research and in critical reviews (e.g. Parsons, 1995; Hoffmann and Parsons, 1997; Spicer and Gaston, 1999; Carey, 2005; Tracy *et al.*, 2006; Chown and Terblanche, 2007), but only recently has the discipline named ‘conservation physiology’ emerged (Wikelski and Cooke, 2006; Cooke and O'Connor, 2010). This discipline has expanded dramatically with the publication of synthetic papers (Carey, 2005; Tracy *et al.*, 2006; Wikelski and Cooke, 2006; Cooke and Suski, 2008; Pörtner and Farrell, 2008), special issues (e.g. Stevenson, 2006; Franklin and Seebacher, 2012), and symposia [e.g. by the Society for Integrative and Comparative Biology (Stevenson *et al.*, 2005; Stevenson, 2006) and Society for Experimental Biology (Franklin, 2009)].

In this overview, we briefly review what we mean by conservation physiology, developing a synthetic definition that reflects the current scope of the field. We then illustrate this scope with a range of examples, demonstrating that conservation physiology has much to offer to science and to those who find themselves with the considerable challenge of practising conservation in our rapidly changing world. In doing so, we provide the reasons for the launch of this new journal, *Conservation Physiology*. Echoing the words of the first editors of *Biological Conservation* (Anonymous, 1968), we offer no excuse for adding another journal to the world's burgeoning journal stable, but rather demonstrate that the need already exists and the time is ripe.

## What is conservation physiology?

‘Conservation physiology’ has been defined previously in several ways, each implying a somewhat different scope. To our knowledge, the first use of the phrase ‘conservation physiology’ was by Wikelski and Cooke (2006), but several earlier phrases were conceptually similar. ‘Conservation physiology’ is more than the sum of its constituent words, though these words are powerful. Conservation is a movement or discipline focused on natural resource use, allocation, and protection (Warren and Goldsmith, 1983; McCormick, 1991; Soulé, 1985). Physiology is focused on the mechanisms involved in how an organism works, including the anatomy and structure of organisms and organs, resource acquisition, metabolism and energy fluxes, regulation and homeostasis, acclimatization to changing environments and environmental tolerances, performance (such as growth, locomotion, and reproductive fitness), and impacts on the ecosystem (Prosser and Brown, 1950; Nobel, 1983; Taiz and Zeiger, 1991; Spicer and Gaston, 1999; Randall *et al.*, 2001). Physiology can be studied at the wide range of scales, from organisms, down to organ systems, organs, cells, and biomolecules and their chemical and/or physical functions.

Carey (2005) suggested that studies can be described as ‘conservation physiology’ when physiologists contribute knowledge, concepts, and perspectives to conservation decision-making. Such a definition is broad, but did not include a statement of the meaning of ‘conservation’. Although a zoologist, Carey (2005) reminded readers of the threats that plants face given environmental change and pathogen emergence. In a paper titled ‘The importance of physiological ecology in conservation biology’, Tracy *et al.* (2005) provided a case study on the nutritional ecology of desert tortoises and noted that ‘many of the threats to the persistence of populations of sensitive species have physiological or pathological mechanisms, and those mechanisms are best understood through the inherently integrative discipline of physiological ecology’. The authors noted that physiological ecology has become critical for understanding threats to the persistence of sensitive species and that physiological data can be used as part of ‘informed opinion’ (ideally, with theory and experiments to test hypotheses that form new syntheses of physiological and ecological knowledge), and eventually to guide management decisions.

Wikelski and Cooke (2006), both zoologists, coined the term ‘conservation physiology’ in a paper with that title. They explicitly defined it as ‘the study of physiological responses of organisms to human alteration of the environment that might cause or contribute to population declines’. The authors acknowledged that conservation physiology was relevant to all taxa, but their examples were entirely focused on vertebrates. One of the greatest limitations of that definition was its being framed only in terms of identifying problems rather than developing solutions. Also limiting is the focus on population declines as an end-point, which fails to recognize other relevant responses, such as range shifts, changes in genetic diversity, and alteration in the structure and function of populations, communities, and ecosystems. Although the authors did not provide a definition of conservation *per se*, they did provide a specific conservation-oriented metric (i.e. population decline), which is the basis for most regional, national, and international threat assessments (e.g. IUCN Red List; Mace *et al.*, 2008). The definition provided by Wikelski and Cooke (2006) remained unchanged for some time despite its limited scope.

Parallel developments were also focusing on the role of physiological studies in understanding the responses of organisms to anthropogenic change, the consequences thereof, and the scope for both mitigation of and adaptation to impacts. In particular, macrophysiology, defined as ‘the investigation of variation in physiological traits over large geographical and temporal scales and the ecological implications of this variation’ (Chown *et al.*, 2004a), had focused on these conservation-related questions, as made explicit in a series of reviews (Chown and Gaston, 2008; Gaston *et al.*, 2009). Subsequent macrophysiological work identified previously unappreciated conservation problems, such as climate-change-related threats to tropical and sub-tropical populations (Deutsch *et al.*, 2008; Huey *et al.*, 2009; Clusella-Trullas *et al.*, 2011; Duarte *et al.*, 2012; Kellermann *et al.*, 2012), illustrating its close links to conservation physiology (see also Chown and Gaston, 2008).

Following a brief hiatus in the discussion of conservation physiology, Seebacher and Franklin (2012) presented a revised definition; one that combined elements of earlier papers (e.g. Carey, 2005; Tracy *et al.*, 2005) with that of Wikelski and Cooke (2006). Conservation physiology became ‘the application of physiological theory, approaches and tools to elucidate and address conservation problems with the aim to provide a mechanistic understanding of how environmental disturbances and threatening processes impact physiological responses and thereby ecological function, population persistence, and species survival’. This definition was the first to use the word ‘mechanistic’, to refer to theory, approaches, and tools, and to expand the scope from populations to include a higher level of biological organization, namely ecological function and species survival. A key point in that paper was the value of conservation physiology in elucidating the ‘cause and effect’ that underpinned environmental impacts on organisms. This definition was taxon neutral, but there was little formal consideration of plants.

In the same year, in a ‘conservation in practice’ paper focused on Pacific salmon, Cooke *et al.* (2012) adopted the definition of Wikelski and Cooke (2006), but showed that physiological knowledge has already been used not only to document problems, but also to generate management models to predict how organisms will respond to change, and to develop and test conservation strategies to generate desirable conservation outcomes (e.g. increases in population size, enabling sustainable use). A range of other studies has also demonstrated the specific conservation and management utility of physiological knowledge (e.g. Porter *et al.*, 2000; Pimm and van Aarde, 2001; Mitchell *et al.*, 2008; Morris *et al.*, 2011; Crossland *et al.*, 2012). Collectively, these examples show that conservation physiology can do more than document problems and elucidate mechanisms, which although important, are not always enough to assist population recovery. Cooke *et al.* (2012) also noted that although conservation physiology is typically focused on anthropogenic stressors, rarely do stressors act alone, and stressors such as diseases can be moderated by human activities, consistent with ideas presented by Carey (2005). Metcalfe *et al.* (2012) revised the previous definitions as follows: ‘We consider conservation physiology to be an applied subdiscipline within ecophysiology and define conservation physiology as the study of physiological responses of organisms to environmental changes and human-induced impacts, and their implications for population and ecosystem dynamics’. Their refined definition explicitly recognized that natural variations in the environment may alter the response of organisms to human impacts. A similar consideration also exists in the field of toxicology, where prior exposure to a toxicant can alter subsequent exposures, e.g. the induction of metallothionein-binding proteins in blood and detoxification enzymes in the liver. They also expanded the definition beyond population declines to ecosystem processes and emphasized scaling from physiological effects to community and ecosystem processes, though missing the overlap with macrophysiology. The authors noted that ‘conservation’ included conservation of biodiversity as well as the management of exploited living resources (presumably in a sustainable manner; Aplet *et al.*, 1992). Metcalfe *et al.* (2012) used marine fish as a case study, but their definition certainly did not exclude plants. However, like previous definitions, they did not focus on the need for this science to solve conservation problems.

As evident from these previous definitions, the concept of ‘conservation physiology’ has evolved. We have reviewed the strengths and weaknesses of the previous definitions and considered additional properties required to embody the actual and potential capabilities of conservation physiology, as follows:

- Taxonomic inclusiveness.
- Interpretation of ‘physiology’ in the broadest sense to include functions and mechanisms at all scales, from the cellular to the organismal, to the community, ecosystem, and biosphere.
- Interpretation of ‘conservation’ in the broadest sense to include conservation of biodiversity as well as the sustainable management of biological resources (Aplet *et al.*, 1992).
- Inclusion of work not only on declines in populations of species of concern, but also on the broad range of problems facing conservation and management, e.g. how to control invasive species, how to maintain habitat, and how to manage fragmented and degraded systems.
- Recognition of the need to scale from physiological mechanisms and processes to the levels of interest to conservation practitioners (e.g. populations, species, communities, ecosystems).
- Focusing not only on the documentation and clarification of conservation problems, but also on identification and refinement of solutions.
- Extending from the core of basic knowledge to improve understanding and inform decision-making in a number of ways, such as incorporation of knowledge into models and other decision-support tools.
- Integration with the wide range of scientific sub-disciplines for maximal power and understanding.

Taking all these elements on board, we arrived at the definition of conservation physiology that is the epigraph of this paper. Consistent with this definition, we have outlined a series of topics that exemplify what conservation physiology encompasses (Box 1).

## The need for conservation science

From the days of Aldo Leopold (1920–40s) and his promotion of the land ethic (in *A Sand County Almanac*) and wildlife management (in *Game Management*) to Rachel Carson's candid assessment of the effects of pesticides on birds, the fate of the natural world and the recognition that we need to take better care of it and even attempt to repair the damage done has become a prevailing societal paradigm. How to do so, of course, remains a considerable challenge given increasing demands on the natural world (Vitousek *et al.*, 1997). The global human population continues to expand rapidly, as does demand for resources. The human population has already doubled in the lifetime of one of the authors and is expected to exceed 9 billion by 2050. However, population size is not the sole problem, because affluence (or consumption) and technology also influence the relationship between humans and the environment (Ehrlich and Ehrlich, 1970; Arrow *et al.*, 2004). The actions and inactions of humans (e.g. Foley *et al.*, 2005; Goudie, 2005) have led to widespread disturbance and environmental change, from local to global scales. In some cases, conservation problems have arisen from intensively managed and exploited species (e.g. Atlantic cod), such that what was once a management issue has since become a conservation problem (Hutchings and Myers, 1994). However, at the same time excellent targeted conservation actions have brought species back from the brink of extinction (Sodhi *et al.*, 2011), fuelling optimism that the future of conservation is less bleak once we understand the mechanistic connections between and within organisms (Gillson *et al.*, 2013).

Although human impact on the environment can be measured in many ways, loss of biodiversity is commonly understood to be important. Biodiversity, in its most simple form, is the variety (at all scales) of life on earth (Magurran, 2004; Millennium Ecosystem Assessment, 2005; Maclaurin and Sterelny, 2008), and is by most accounts in decline (Dirzo and Raven, 2003). A recent report revealed that despite some local successes, and considerable efforts to slow the decline, the rate of biodiversity loss is not slowing (Butchart *et al.*, 2010). Amphibians (Stuart *et al.*, 2004; Hof *et al.*, 2011), freshwater fish (Ricciardi and Rasmussen, 2001), and tropical forests, as well as their resident biota (Wright, 2005), show particularly high rates of biodiversity loss and extinction. Loss of biodiversity is often first noted as declines in population sizes, a common metric used for regional, national, and international threat levels (Rodrigues *et al.*, 2006). Population declines are pervasive, with examples in nearly every region and taxon (e.g. sea grasses, Orth *et al.*, 2006; coral reefs, Pandolfi *et al.*, 2003). Vertebrate imperilment status (as measured by the IUCN Red List) continues to be dire, although the deterioration in species status would have been worse in the absence of conservation efforts (Hoffman *et al.*, 2010). Alarmingly, declines in the abundance of species and the loss of biodiversity have direct and indirect effects on human well-being (Diaz *et al.*, 2006; Hanski *et al.*, 2012). Although there is some debate regarding the ecosystem consequences of biodiversity loss (e.g. Naeem, 2002), in general it is recognized that there is real potential to alter ecosystem properties and the goods and services they provide to society (Hooper *et al.*, 2012). Effective conservation of biodiversity is essential for human survival and the maintenance of ecosystem processes and services (Rands *et al.*, 2010).

**Box 1: The scope of conservation physiology**

- Understanding the influences of anthropogenic disturbance and variation in habitat quality on organism condition, health and survival
- Providing a mechanistic/functional understanding of the effect of anthropogenic environmental change on organisms; using physiological knowledge to develop mechanistic models for species distributions
- Evaluating stress responsiveness and environmental tolerances relative to environmental change (including global climate change and ocean acidification)
- Developing mechanistic relationships between population declines and physiological processes
- Understanding the relevance of acclimatization and adaptation of physiological processes to environmental variation (e.g. studies of thermal adaptation among populations and species) to management and conservation
- Understanding the physiological mechanisms involved in changes in community, ecosystem and landscape structure, as well as individual species, in response to environmental change
- Applications of contemporary genomic and post-genomic technologies to conservation physiology
- Integration of physiology with conservation behaviour, conservation medicine, conservation toxicology, conservation genetics, and other relevant sub-disciplines (Table 1)
- Understanding the relevance of ecology and evolution of physiological diversity to conservation
- Exploiting knowledge of organismal physiology to control invasive species and restore threatened habitats and populations
- Understanding the optimal environmental conditions for *ex situ* preservation of endangered species (captive breeding, seed bank protocols for storage and regeneration, tissue culture for plant species or genotypes that are difficult to regenerate from seeds)
- Evaluating and improving the success of various management and conservation interventions
- Applying physiological biomarkers as part of long-term environmental monitoring programms
- Developing predictive models in conservation practices that include physiological parameters
- Integrating physiological knowledge into ecosystem management and development of tools to solve complex conservation problems
- Understanding the policy implications of conservation physiology research

In the 1980s, scientists such as Michael Soulé began to consider the need for a new multi-disciplinary field dedicated to the science of scarcity and diversity (Soulé, 1986). Conservation biology was born, with a focus on providing principles and tools for preserving biological diversity (Soulé, 1985). The field matured and evolved, and eventually adopted an even more holistic perspective and moniker (i.e. conservation science). This field contributed new approaches to conservation practice, such as conceptual frameworks (e.g. Salafsky *et al.*, 2002; McGeoch *et al.*, 2006, 2012; Pereira *et al.*, 2013) for scientists to provide information relevant to conservation practitioners (e.g. managers, policy-makers). The recognition grew that evidence, rather than anecdote, was essential for meaningful conservation action (Sutherland *et al.*, 2004), and especially to move beyond arbitrary or *ad hoc* approaches (Pullin *et al.*, 2004).

Within the sphere of conservation science several sub-disciplines have emerged, including conservation genetics, conservation medicine, and conservation social science (see Table 1). Many of these sub-disciplines have become recognized specialty areas in their own right, beyond their integration within the broader realm of conservation science. All of these sub-disciplines are focused on generation of knowledge to understand problems as well as to develop solutions. Indeed, modern conservation science is as much about restoring ecosystems and rebuilding populations (e.g. Hobbs and Harris, 2001) as it is about documenting responses to stressors. The exception, perhaps, is in terms of predicting the future (e.g. Pearson and Dawson, 2003; Bellard *et al.*, 2012; Huey *et al.*, 2012) such that it is possible to develop adaptation strategies for environmental change. Clearly, given the multitude of threats facing biodiversity, population persistence, and ecosystem structure and function, conservation science plays an essential role. Likewise, resource management, even of exploited populations, has at its heart conservation and the science behind it (see Noss, 2006; note that this was also recognized by Anonymous, 1968), because management activities were intended to be based on sustainable use (Rice *et al.*, 1997; Martinet *et al.*, 2007) within an ecosystemic context (Grumbine, 2002). International policy instruments, laws, agreements, and initiatives, such as the Convention on Biological Diversity, the Convention on the International Trade of Endangered Species, and the Millennium Ecosystem Assessment and Development Goals, provide institutional guidance for addressing some of the larger-scale conservation issues, although in reality most successes have been based on more localized activities.

**Table 1:** Summary of the list of the various sub-disciplines of conservation science with relevant connections to conservation physiology

Sub-disciplines	Summary of sub-discipline and key references	Examples of potential integration with conservation physiology
Conservation anthropology	Documenting knowledge, traditions, concerns and definitions of different stakeholders relative to conservation (Orlove and Brush, 1996; Brosius 2006)	Knowledge on the physiology of native organisms can be extracted from stakeholders, providing direction for experimentation or further investigation (e.g. for rainforest conservation; Ellen, 1997)
Conservation behaviour	Understanding behavioural variation and exploiting it to develop tools for preventing extinction (Sutherland, 1998; Caro, 1999; Buchholz, 2007; Ruxton and Schaefer, 2012)	Physiology has the ability to elucidate mechanisms associated with alterations in behaviour
		Physiology and behaviour yield a more complete understanding of individuals, and how different drivers could scale up to affect higher levels of biological organization
		Integration could improve predictions of individual responses to environmental perturbations (based on exposure and sensitivity; Metcalfe *et al.*, 2012)
		Integration could be particularly relevant for *ex situ* conservation and issues associated with captive breeding and reintroductions
		Quantifying secondary impacts on plants of threats to animal pollinators and dispersers
Conservation biogeography	Application of concepts and methods of biogeography to address conservation problems and to provide predictions about the fate of biota (Simberloff and Abele, 1976; Richardson and Whittaker, 2010)	Knowledge of variation in physiological traits over large geographical, temporal, and phylogenetic scales can contribute to addressing how drivers of environmental change operate (Chown and Gaston, 2008)
Conservation ethics	Consideration of the ethical dimension of conservation, natural resource management, and sustainability (Callicott, 1991, 2005)	Physiology could be used to resolve questions regarding what the appropriate measures of ecosystem integrity or health may be
Conservation genetics and genomics	Conservation of genetic diversity and the application of genetic and genomic methods towards resolving problems in conservation (Frankham, 1995; Hedrick, 2001; Frankham *et al.*, 2002; Ryder, 2005; Kramer and Havens, 2009; Primmer, 2009)	Could be used to understand and define discrete conservation units/populations/stocks that can be evaluated for physiological capacity and tolerances to characterize the consequences of such genetically based categorizations
		Physiology can be used to assess the consequences of outbreeding and inbreeding depression on organismal fitness
		Use of molecular tools (e.g. gene arrays) for assessment of loci or genes that may be directly involved in responses to processes such as environmental change (Ryder, 2005; Primmer, 2009)
		Physiology can be used to improve quantification of functional differentiation among populations, to set priorities
		Physiological knowledge is essential to test hypotheses concerning whether populations are occupying optimal habitats
Conservation medicine	Understanding the relationship between human and animal health (e.g. disease transfer), and environmental conditions to inform conservation (Deem *et al.*, 2000, 2001; Meffe, 2001; Aguirre *et al.*, 2002; Ostfeld *et al.*, 2002; Tabor, 2002; Niinemets and Peñuelas, 2008)	The basis for veterinary and human medicine is organismal anatomy and physiology
		Physiology can identify consequences of disease for organisms and, in some cases, the triggers (e.g. stress)
		Physiology and conservation medicine could be used in parallel to address the causes and consequences of outbreaks of disease and biotoxins (e.g. toxic algal blooms), thus potentially revealing solutions (Aguirre *et al.*, 2002)
		Quantifying the impacts of non-native plants on ecosystem ‘health’ and human health
Conservation planning	Process (ideally systematic) that is defensible, flexible, and accountable to enable plans to be devised and reviewed in order to enable conservation objectives to be met (Groves *et al.*, 2002; Pierce *et al.*, 2005; Margules *et al.*, 2007; Pressey *et al.*, 2007)	Physiological tools can be used as part of monitoring programmes to review successes of plan components
		Physiological knowledge can be used to inform the selection and refinement of action elements of conservation plans (Wikelski and Cooke, 2006)
		Physiology can be used to identify and prioritize threats that would need to be mitigated as part of species or ecosystem recovery plans
Conservation policy	Development of policy instruments and governance structures consistent with the principles of conservation science (Meffe and Viederman, 1995; Ludwig *et al.*, 2001)	Physiology can provide mechanistic explanations and establish cause-and-effect relationships consistent with generating an evidence base to support policy and decision-making (Cooke and O'Connor, 2010)
Conservation psychology	Understanding the reciprocal relationships between humans and the rest of nature, with a particular focus on how to encourage conservation (Bott *et al.*, 2003; Saunders, 2003; Kaufman *et al.*, 2006)	Physiological approaches could identify and clarify processes and mechanisms that could enable stakeholders to make better connections to conservation issues
Conservation social science	Understanding how socio-economic factors (e.g. markets, cultural beliefs and values, wealth/poverty, laws and policies, demographic change) shape human interactions with the environment (Costanza, 1991; Jacobson and Duff, 1998; Mascia *et al.*, 2003)	The cause-and-effect nature of physiology could alter stakeholder perspectives of conservation issues by providing credibility and relative certainty
Conservation toxicology	Understanding and predicting the consequences of pollutants on various levels of biological organization to inform conservation action (Hansen and Johnson, 1999, 2009)	Physiology is a core component of toxicological studies and can be used to identify the mechanisms of action and thresholds for various pollutants (Hansen and Johnson 1999, 2009)
		Physiology can be used to inform risk assessments and support regulatory processes related to pollution
Landscape ecology	Understanding and improving relationships between ecological processes in the environment and particular ecosystems (Hansson and Angelstam, 1991; Hobbs, 1997; Gutzwiller, 2002)	Physiological indices have the potential to contribute to understanding of how landscape pattern affects persistence of populations and species (Chown and Gaston, 2008; Ellis *et al.*, 2012)
		Physiological tools could indicate problems with habitat quality before it is manifested in negative consequences at the population level (i.e. early warning system; Cooke and Suski, 2008; Ellis *et al.*, 2012)
		Physiology would clarify the cause-and-effect relationship that links landscape change to population responses (Ellis *et al.*, 2012)
Natural resource and ecosystem management	Managing the way in which people and natural resources interact to maintain ecosystem services, including sustainable human use (Ludwig *et al.*, 1993; Grumbine, 2002; Hawthorne *et al.,* 2012)	Physiology can be used to determine whether management actions are themselves causing problems by monitoring organismal condition and stress (Wikelski and Cooke, 2006)
		Can be used to identify best practices for management actions of direct relevance to stakeholders (e.g. bycatch reduction strategies, reforestation)
		Physiology can inform decision-support tools/models (see above)
Population and ecosystem biology and modelling	Application of quantitative modelling techniques to characterize and predict population, community, and ecosystem dynamics relative to stressors and conservation actions (Simberloff, 1988; Beissinger and Westphal, 1998; Schwartz *et al.*, 2000; Medvigy and Moorcroft, 2012)	Physiological knowledge can be incorporated into ecological models to improve their reliability and accuracy (Metcalfe *et al.*, 2012)
		Physiology can provide the basis for understanding demographic change by linking organismal performance (e.g. growth, fitness) to environmental conditions (Ricklefs and Wikelski, 2002; Young *et al.*, 2006)
		Models provide decision-support tools for practitioners that enable physiological data to be scaled up to be relevant to ecological processes
		Physiology can experimentally validate models
		Potential to generate mechanistic predictive models of how organisms respond to climate change (Pearson and Dawson, 2003)
Restoration science	Practice of renewing and restoring degraded, damaged, or destroyed ecosystems and habitats in the environment by active human intervention and action (Dobson *et al.*, 1997; Young, 2000; Giardina *et al.*, 2007)	Physiological knowledge (e.g. environmental tolerances of plants) can be used to inform the selection of candidate taxa to be used in restoration and remediation activities (Pywell *et al.*, 2003; Ehleringer and Sandquist, 2006; Cooke and Suski, 2008)
		Physiological tools can be used to monitor the success of restoration activities (Cooke and Suski, 2008)
		Physiological knowledge can be exploited to inform the control of invasive or introduced species (e.g. Wagner *et al.*, 2006)

Sub-disciplines are listed alphabetically.

## The need for conservation physiology

**Figure 1:**
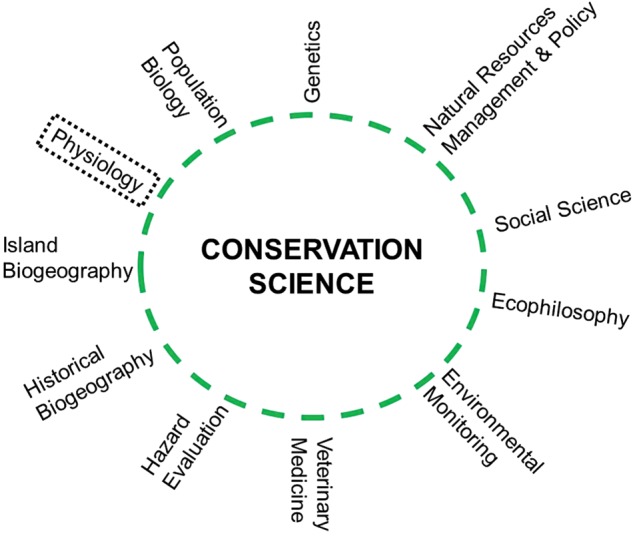
Components of conservation science [Soulé (1985); referred to as conservation biology]. Note how physiology is included as a component even in this early (Soulé, 1985) schematic diagram despite the fact that some other prominent sub-disciplines (e.g. behaviour, planning; see Table 1 for complete list) are excluded. Republished with permission of The American Institute of Biological Sciences, from Soulé (1985; BioScience). Permission conveyed through Copyright Clearance Center, Inc. (Detail ID 63243232, Licence ID 3051560892668).

Conservation physiology is a latecomer relative to the other sub-disciplines that have become trusted and recognized components of conservation science (e.g. conservation genetics, conservation behaviour). While physiology was identified as relevant (see Fig. 1; Soulé, 1985) in the early days of conservation biology, one must look quite hard to find even a mention of physiology in any of the introductory conservation biology textbooks (e.g. Primack, 1993; Pullin, 2002; Lindenmayer and Burgman, 2005; Groom *et al.*, 2006; Hunter and Gibbs, 2006). It is almost as if the relevance of physiology was forgotten or dismissed for some time. Fazey *et al.* (2005) evaluated publication trends in conservation science and revealed that most research was focused on species and populations, rather than the broader suite of scales from molecules to ecosystems, demonstrating that physiology was poorly represented within at least key conservation science journals. In the field of ecological restoration, some have even expressed doubt that a general mechanistic approach is necessary at all (e.g. Cabin, 2007), while others have vigorously argued that it is essential (e.g. Giardina *et al.*, 2007; Valladares and Gianoli, 2007; Cooke and Suski, 2008; Brudvig, 2011). We believe that this oversight, intentional or not, reflected the difficulty of easily connecting physiology with its application. With the expanded knowledge in the mechanistic aspects of environmental physiology and environmental toxicology, this is no longer the case. The growth of physiology back towards ecology and application to conservation has been deliberate, albeit perhaps somewhat stealthy.

Why is conservation physiology needed? In answering this question, we must go beyond the bland statement that linkages between physiology and conservation have become possible. Therefore, more specifically, what does conservation physiology contribute to conservation science, resource management, and policy? In fact, many roles can be outlined for the importance of conservation physiology within the broader sphere of conservation science. These roles can be targeted specifically towards current needs, as recommended by Noss (2006). Indeed, there have been several syntheses that have outlined priority research topics (via horizon scanning and collaborative prioritization exercises) and needs in conservation science in general (see Soulé and Orians, 2001; Sutherland *et al.*, 2009, 2010, 2011, 2012), as well as for specific regions (e.g. the UK, Sutherland *et al.*, 2006; USA, Fleishman *et al.*, 2011; Canada, Rudd *et al.*, 2011; Antarctica, Chown *et al.*, 2012a), taxa (e.g. turtles, Hamann *et al.*, 2010; birds, Sutherland *et al.*, 2012; insects, Stewart, 2012; pollinators, Dicks *et al.*, 2013; metallophytes, Whiting *et al.*, 2004), and pressing problems (e.g. pharmaceuticals in the environment, Boxall *et al.*, 2012; coastal and marine management, Rudd and Lawton, 2013). Such research priority documents can be mined to identify potential ways in which conservation physiology can be relevant to practitioners. For example, in the 2012 horizon scan of emerging global conservation issues, Sutherland *et al.* (2012) listed a number of issues that are in desperate need of mechanistic physiological studies to understand and solve problems (e.g. warming of the deep sea, mining in the deep ocean, climate-driven colonizations in Antarctic waters, increases in pharmaceutical discharges as human populations age, and the potential consequences of graphene on organisms). In addition, a group of physiologists (i.e. Tsukimura *et al.*, 2010) suggested that physiology had the potential to inform and address a variety of conservation issues and emphasized the need to transform such science into information and tools that other constituencies can process and use. Clearly, there is no shortage of opportunity for the nascent field of conservation physiology.

It is not possible to review all of the possible applications here, so we refer the reader to other syntheses, including Carey (2005), Wikelski and Cooke (2006), Chown and Gaston (2008), Cooke and O'Connor (2010) and Seebacher and Franklin (2012), as well as to Table 2, for examples of the potential ways in which various research areas within physiology can contribute to plant and animal conservation physiology and conservation science. For all examples provided, a core strength of physiology, i.e. the mechanistic approach (Carey, 2005; Cooke and O'Connor, 2010), is to identify and confirm cause-and-effect relationships through experimentation. Indeed, Carey (2005) suggested that physiologists could be helpful in setting standards for the type of evidence that would constitute compelling proof of a cause-and-effect relationship. Given that physiology moves beyond correlation to seeking causation (Seebacher and Franklin, 2012), it arrives at robust information that would be compelling in legal proceedings, such as are common for conservation and environmental issues (e.g. De Klemm and Shine, 1993), as well as toxicological ones.

**Table 2:** Examples of physiological sub-disciplines and their potential contributions to conservation of animals and plants (modified and expanded from Wikelski and Cooke, 2006)

Physiological sub-discipline	Potential contributions to animal conservation	Potential contributions to plant conservation
Bioenergetics and nutritional physiology	Provides opportunity to measure organismal condition and energy allocation relevant to growth and reproduction (Stevenson, 2006)	Understanding how plant species, communities, and biomes impact on climate and atmospheric composition, and how they respond to climate change (Hepburn *et al.*, 2011; Lohbeck *et al.*, 2012)
	Details the nutritional needs, state, and deficiencies of animals in the wild and in captivity to identify problems (Tracy *et al.*, 2005)	Provides a quantitative basis for the conservation of species and ecosystems globally (Vitousek, 1994)
	Provides the knowledge needed to sustain animals in captivity and provide them with necessary resources to reproduce (Saint Jalme, 2002)	Provides a quantitative basis for preventing the spread of invasive species and degradation of landscapes and to prioritize restoration (Vitousek and Walker, 1989)
Cardiorespiratory physiology	Informs animal–environment relationships, given that respiratory capacity constrains organismal performance (Farrell *et al.*, 2009)	Not applicable
	Enables development of aerobic scope models to predict animal responses to environmental change (Farrell *et al.*, 2008; Eliason *et al.*, 2011)	
Chemical communications (i.e. endocrinology and plant growth regulators)	Enables the assessment and quantification of stressors that can ultimately affect fitness or survival (Busch and Hayward, 2009)	Plant growth regulators allow artificial control of reproduction to improve germination and outplanting (e.g. Sarasan *et al.*, 2006).
	Provides tools for evaluating strategies for ameliorating or minimizing stress	Facilitates the chemical control of weeds and herbivores (Tu *et al.*, 2001)
	Provides information about the reproductive biology of organisms that can be used for captive breeding or biological control (Stevenson *et al.*, 2005)	
Comparative physiology and biochemistry	Develops generalizations and relationships that can be used in predictive capacities (Wikelski and Cooke, 2006)	Allows quantitative characterization of distinct populations and species (Hartmann, 1996)
	Provides tools for examining how different species and populations respond to different stressors	Allows quantification of baseline physiology to allow rapid determination of stress responses (Jackson, 1986)
		Develops generalizations and relationships that can be used in predictive capacities
Environmental and ecological physiology	Enables understanding of the distribution and abundance of different organisms in different environments based on environmental tolerances (Spicer and Gaston, 1999)	Enables understanding of the distribution and abundance of different organisms in different environments based on environmental tolerances (Larcher, 2003; Lambers *et al.*, 2008)
	Elucidates the responses of organisms to environmental change and the development of predictive models (Pörtner and Farrell, 2008; Buckley *et al.*, 2011; Franklin and Seebacher, 2012)	Elucidates the responses of organisms to environmental change and the development of predictive models (Nicotra *et al.*, 2011)
Environmental toxicology	Provides information about the physiological effects of different environmental contaminants on organisms (Hansen and Johnson, 1999, 2009)	Understanding and alleviating environmental stresses on plants (Marrs *et al.*, 1989)
	Enables the assessment of strategies (e.g. regulatory guidelines) for minimizing those effects	Understanding how plants may be used for remediation of contaminated landscapes (Marmiroli *et al.*, 2006)
	Mechanistic explanations of sub-lethal metal toxicity in fish	Understanding tolerance of grasses to high metal concentrations in soils near mines
Evolutionary physiology	Provides information about the factors that guide, direct, and constrain physiological evolution (Garland and Carter, 1994)	Provides information about the factors that guide, direct, and constrain physiological evolution
	Links directly to the life history and, thus, population biology and fate of organisms	Links directly to the life history and, thus, population biology and fate of organisms (Edwards, 2006)
	Develops models to predict the long-term evolutionary consequences of selection for different phenotypes	Develops models to predict the long-term evolutionary consequences of selection for different phenotypes (Kharouba *et al.*, 2012)
		Determination of the degree that tolerance and plasticity can match that of environmental change, and how populations are likely to shift in their distributions (Thuiller *et al.*, 2008)
Immunology and epidemiology	Provides an understanding of the effects of immune disorders and disease on organismal performance and survival (Stevenson *et al.*, 2005)	Provides an understanding of the effects of disease and disease resistance on organismal performance and survival (Anderson *et al.*, 2004)
	Aids in understanding pathogen behaviour and consequences, which is particularly important for conducting population viability analysis of stressed or rare organisms (e.g. Blaustein *et al.*, 2012)	Provides opportunity to refine strategies for surveillance and control of diseases (Anderson *et al.*, 2004)
Locomotor performance physiology	Provides understanding of whole-organismal performance, through measures of locomotor activity, and maximal performance, which is a proxy for fitness (Brauner *et al.*, 2012)	Not applicable
Neurophysiology and sensory biology	Facilitates understanding of the neural basis of behaviours, which is important because a fundamental understanding of conservation-related animal behaviour has been repeatedly identified as an essential prerequisite for biological conservation (Cockrem, 2005)	Not applicable
	Provides information on organismal sensory physiology that can be exploited to manipulate animal behaviour for conservation purposes (e.g. development of deterrents for interacting with human infrastructure or activities; Southwood *et al.*, 2008)	
Physiological genomics	Details the functioning of gene products in the context of the whole organism and its environment (Ryder, 2005)	Details the functioning of gene products in the context of the whole organism and its environment (Ryder, 2005). Towards rapid characterization of differences among populations in ecological tolerances, as done for crop varieties (Mir *et al.*, 2012)
	Reveals information that can be used to understand how organisms will respond to environmental change and for characterization of molecular physiological diversity (Miller *et al.*, 2011)	Reveals information that can be used to understand how organisms will respond to environmental change and for characterization of molecular physiological diversity
Reproductive physiology	Provides information about the control and regulation of reproduction, the influence on sex cell production and maturation, and ultimately, measures of fecundity, which are a proxy for fitness	Quantification of optimal range of conditions to induce flowering, maximize pollination, germinate seeds, and establish and maintain field populations (Hegland *et al.*, 2008)
		Predicting the effects of environmental change on species and vegetation system succession and regeneration (Cheaib *et al.*, 2012)

Sub-disciplines are listed alphabetically.

Here we provide a brief overview of key ways in which conservation physiology can contribute in an important manner to conservation science. As noted by Cooke and O'Connor (2010), there remain challenges in ensuring that conservation physiology is relevant to policy-makers and conservation practitioners, and thus we aim to demonstrate with concrete examples where successes have been achieved in conservation physiology (see Cooke *et al.*, 2012).

### Characterizing physiological diversity, its ecological implications, and importance for conservation

Physiological diversity is the variation in function and tolerances among individuals, populations, and species, and arises due to a combination of genetic, developmental, and environmental influences (Feder, 1987; Spicer and Gaston, 1999; Chown, 2012). At the most basic level, physiological assessments can identify and characterize functional biodiversity, a necessary requisite to biodiversity conservation. For example, substantial efforts in characterizing physiological diversity in insects (reviewed by Chown and Terblanche, 2007) have revealed that as a consequence of limited upper thermal tolerances and the form of the metabolic rate–temperature relationship, tropical and sub-tropical populations are likely to be much more at risk from changing climates than their more temperate counterparts (Deutsch *et al.*, 2008; Dillon *et al.*, 2010; Kellermann *et al.*, 2012; Hoffmann *et al.*, in press). Likewise, it appears that responses to changing environments might differ substantially between the hemispheres (Chown *et al.*, 2004b), and that winter warming may be especially problematic for temperate species (Williams *et al.,* 2012). In this respect, Beardall *et al.* (1998) point out that the austral marine flora may be more sensitive to global warming than their boreal counterparts. Algal reproduction is extremely sensitive to temperature and, for instance, Breeman (1990) predicted significant changes, due ocean warming, in coastal community structure associated with the northward shift in the southern boundaries of the major canopy-forming kelps of the genus *Laminaria*.

Understanding what such diversity means for the organization and function of ecosystems is also a growing and necessary topic (Pachepsky *et al.*, 2001). The recognition that inter-individual variation in physiological diversity can generate intra-population diversity has important implications for conservation (e.g. population reintroduction programmes). Several approaches to explain broad-scale patterns in biodiversity have incorporated elements of physiological diversity (e.g. Chown *et al.*, 2002; Gaston, 2003; Buckley *et al.*, 2012). As noted by Spicer and Gaston (1999), attempts to derive such relationships between physiological diversity and distribution and/or abundance should be based, where possible, on hypothesis-driven manipulative examinations, not only to document patterns, but also to understand their mechanistic basis. Such examples now exist. For instance, Navas (2002) used both ecological and evolutionary physiology to document herpetological diversity along altitudinal gradients in the Andes. Likewise, greater animal diversity at cold seeps vs. hydrothermal vents appears to be related to the greater physiological barriers to invasion of hydrothermal vents (Turnipseed *et al.*, 2003). As shown for Hawaiian *Plantago* species, studies of the physiology of rare plant species within a genus can indicate traits that make them distinctive as conservation priorities, and additionally, reflect their differentiated habitat preferences (Dunbar-Co *et al.*, 2009).

### Identifying critical habitats and understanding the consequences of variation in habitat quality

Habitat is the foundation of functional ecosystems. It is therefore not surprising that many conservation and management efforts relate to identifying and protecting critical habitats from human alteration (Hagen and Hodges, 2006). Studies of animals have used behavioural information to study their spatial ecology; however, there is increasing interest in documenting the fitness benefits of occupying different habitats and niches, particularly in a bioenergetics context. Niche theory, encompassing both the fundamental niche, where species are able to survive, and the realized niche, where species are actually found, provides a useful framework to examine the influence of abiotic and biotic factors on the distribution of organisms and to predict the impact of environmental change (Hutchinson, 1957; Kearney and Porter, 2004; Holt, 2009). Physiology plays a fundamental role in setting a fundamental niche, and a secondary role to expressed behaviours in setting a realized niche (Huey, 1991). Without such physiological knowledge, therefore, niche descriptions become limited (Porter and Tracy, 1983). Furthermore, Wilson *et al.* (2012) described the concept of ‘energy landscapes’ as a means to consider how animals may make decisions about how they select various habitats or movement paths. Given that landscapes and their constituent habitats vary in composition, such habitats will be of different value to different taxa. In some cases, organisms will be excluded due to environmental thresholds that exceed their tolerances (e.g. light, temperature, moisture), but in other cases organisms may persist in areas of lower habitat quality although they suffer fitness consequences for doing so (Huey, 1991). Thus, for rare species, as well as for populations of common species at the edge of their ranges, and subject to the influence of climate change, detailed quantification of habitat and physiological responses is necessary (Aleric and Kirkman, 2005; Liu *et al.*, 2006). Indeed, Oftedal and Allen (1996) advocate ensuring that the nutritional requirements for reptiles are met within protected habitats if they are to benefit reptile conservation. In one study, estuarine habitat quality was evaluated using fish condition as a proxy (Amara *et al.*, 2009). Conservation physiology can also be used to inform habitat-related management actions. For example, Hasler *et al.* (2012) evaluated energy use in endangered salmon during their spawning migration in different reaches of a regulated river to identify areas where energy use was elevated and thus could represent areas in need of restoration as well as to inform minimal flows. In a unique case, information on the thermal physiology of an endangered Australian snake was used to predict its critical habitat needs from a thermoregulatory perspective (Webb and Shine, 1998). Given that protecting all habitats is unrealistic, physiological tools could be useful for identifying areas that are functionally (rather than structurally) important and that serve as critical habitats.

### Predicting how organisms will respond to environmental change

Changes in the abiotic environment affect the physiology of organisms at multiple levels, which is problematic given the level of anthropogenically mediated environmental change currently underway. From ocean acidification to global climate change and from the Arctic to the Amazon, we need to predict how organisms will respond to such changes. Hepburn *et al.* (2011) suggested that increased growth and competitive ability of non-calcareous marine macroalgae, alongside negative impacts of acidification on calcifying species, could have major implications for the functioning of coastal reef systems at elevated CO_2_ concentrations. Studies on microalgae, with their short generation times, have provided insights regarding adaptive responses to global change (see Lohbeck *et al.*, 2012). Human ‘adaptation’ or adjustments to resource availability and risk are necessary to protect livelihoods (Adger *et al.*, 2005; Smit and Wandel, 2006). The perceived importance of environmental change and the associated motivation for human response depends largely on the rate and magnitude of environmental change and the projected degree to which humans will be affected (Smithers and Smit, 1997; Dawson *et al.*, 2011). Physiological approaches can be used in experimental tests of the response of individual organisms to different types of environmental change (individual and multiple stressors), thereby enabling predictions for future environmental scenarios (Pörtner and Farrell, 2008; Pörtner, 2008; Chevin *et al.*, 2010; Hoffmann and Sgrò, 2011; Huey *et al.*, 2012). For example, Farrell *et al.* (2008) used aerobic scope models and biotelemetry data to predict the success of spawning migrations relative to warming river conditions. Phenotypic plasticity enables the persistence of organisms within a species across a range of environmental conditions to a point; however, it is understood that there is also a limit to physiological compensation for environmental variability such that if conditions exceed the tolerances or capacities of a species, it will be extirpated from a given location (Seebacher and Franklin, 2012). As reviewed by Seebacher and Franklin (2012) and Nicotra *et al.* (2011), compensatory responses occur at different time scales, including between generations (genetic adaptation) and during development (developmental plasticity) so that phenotypes are matched to prevailing environmental conditions, and within the adult lifespan as reversible plasticity (acclimation and acclimatization; Wilson and Franklin, 2002). Individual physiological acclimatization capacity will define the winners and losers relative to different types and extents of environmental change which will be driven (Somero, 2010). Also relevant are the suite of options available to humans in order to respond to environmental change (Smithers and Smit, 1997), something that could be clarified by conservation physiology (e.g. which species are likely to be capable of surviving in a given environment and should we attempt to introduce them to replace the function of species that are extirpated?). In some cases, knowledge of how organisms respond to environmental change could help to identify potential mitigation strategies (Chown and Gaston, 2008).

### Identifying the sources and consequences of different stressors on organisms

Disturbance is pervasive as a result of human activities (Vitousek *et al.*, 1997). Physiological tools can be used to identify the sources and consequences of stressors on plants and animals. Of particular importance is the ability to identify thresholds which either do not elicit stress or which do so at a level that is not maladaptive (Busch and Hayward, 2009). Novel ways now exist to assess stress without having to handle animals repeatedly (e.g. use of faecal glucocorticoid monitoring, biotelemetry, and biologging) in large part due to a desire to apply such measurements to conservation problems. Two of the earliest of such studies on vertebrates showed that logging (Wasser *et al.*, 1997) and snowmobile activity (Creel *et al.*, 2002) can increase glucocorticoid stress hormone release in Spotted Owl and elk (*Cervus canadensis*), respectively, and these discoveries assisted with reserve zoning to restrict such activity in some areas. Similar work has occurred with a range of other taxa and in response to a variety of other stressors (e.g. fisheries interactions, ecotourism, urbanization, aircraft noise). Physiological knowledge has also been used to identify regulatory thresholds for various pollutants for plants (e.g. Das *et al.*, 1997) and animals (Monserrat *et al.*, 2007). Another important aspect of such work has been to identify how stress responses vary relative to differences in habitat quality (e.g. Martínez-Mota *et al.*, 2007; Homyack, 2010), as well as determining when and how such stressors affect fitness and population-level processes (for discussion see Cooke and O'Connor, 2010). Various stressors also have the potential to promote or mediate disease development and, given high-profile problems such as chytrid fungus and amphibians, conservation physiology approaches are being used to understand disease dynamics (Blaustein *et al.*, 2012; Meyer *et al.*, 2012). With human populations expected to continue to grow and our footprint to expand, it is certain that organisms will face more disturbance and pollutants in the future.

### Understanding reproductive physiology to inform *ex situ* conservation activities

*Ex situ* conservation activities related to highly endangered species remain important safeguards for plants (Cohen *et al.*, 1991; Raven, 2004; Pence, 2010) and animals (Balmford *et al.*, 2002). In many ways, such efforts represent the last resort (Philippart, 1995) and are undesirable in that they are resource intensive and expensive, not to mention that there are limitations with such programmes (see Snyder *et al.*, 2002). However, there have been some remarkable successes, and in many of those instances, success has occurred because of a strong understanding of the reproductive biology of the organism (e.g. Sanz and Grajal, 1998; Pukazhenthi and Wildt, 2004). In fact, much of the earliest work in conservation physiology was directly related to reproduction (see Holt *et al.*, 2003). In vertebrates, knowledge of endocrine function is typically exploited to monitor and manipulate the reproductive state of captive animals (e.g. Brown, 2000; Hildebrandt *et al.*, 2007; Schwarzenberger, 2007). In the field of stress physiology and environmental toxicology, the interactions of cortisol and estrogen mimics are well documented at the physiological and biochemical levels, e.g. sex reversal of fishes by estrogen mimics (Jobling *et al.*, 1998). Although many of these efforts are directly related to understanding reproductive function and how to manage and maximize reproductive output, other elements of physiology have proved to be valuable. For example, understanding the nutritional physiology of organisms is key when feeding animals in captivity, to ensure that they have the necessary energy and nutrients to engage in reproduction and produce viable offspring (e.g. Cayot and Oftedal, 1996; Houston *et al.*, 2007). Also important is minimizing stress during captivity and translocation of animals (e.g. Saint Jalme, 2002; Dickens *et al.*, 2009) and ensuring that the appropriate environmental conditions are provided. For plants, knowledge of seed dormancy has enabled the development of seed storage for germplasm conservation (Bonner, 1990), and knowledge of habitat requirements and plant–animal interactions continues to improve the outlook for preserving species from extinction and restoring ecosystems (Giardina *et al.*, 2007; Ruxton and Schaefer, 2012).

### Informing the selection of various conservation actions

Conservation practitioners, resource managers and policy-makers often make decisions regarding conservation actions to undertake in order to address given objectives. They are often guided by various plans (e.g. conservation plans, recovery plans, wildlife management plans, forest management plans; Groves *et al.*, 2002) and the suite of options available to them is ideally based on scientific evidence (Pullin *et al.*, 2004). In addition, actions are ideally implemented in an active, adaptive way such that monitoring will lead to revision of plans and refinement of actions as appropriate (McCarthy and Possingham, 2007). Conservation physiology can be used as an integral part of such monitoring programmes (see below; Cooke and Suski, 2008), and furthermore, to assist the identification of actions likely to be most successful. For example, bycatch reduction strategies for sea turtles have benefited greatly from knowledge of sensory physiology (e.g. to develop repellents; Southwood *et al.*, 2008). In addition, physiological knowledge has been useful in elucidating the chemical ecology of natural enemies, herbivores, and host plants such that biological control programme efforts can focus on the most successful strategy (Khan *et al.*, 2008). In general, however, physiology is perhaps most useful in parameterizing ecological and management models to support decision-making. For example, Metcalfe *et al.* (2012) describe how various models (e.g. population models, individual-based models, species distribution models, and mass- or energy-balanced models) can incorporate information on the relationship between physiology and the environment to inform management actions. As part of a risk assessment, Arriaga *et al.* (2004) used species-distribution models populated with physiological data to assess the invasion potential of buffel grass in Mexico to inform management actions related to preventing such an invasion. Likewise, Chown *et al.* (2012b) used degree-day information for flowering plant species being transported to Antarctica, along with explicit information on visitor numbers and current and future climates to predict areas of most risk for the establishment of invasive alien species. These assessments are now directing conservation management in the region. Arriaga *et al.* (2004) emphasized the need for ecophysiological experiments to improve the precision of such models. As modelling techniques become more sophisticated, physiology will play an important role in ensuring that these decision-support tools are appropriately parameterized and calibrated through careful experimentation.

### Evaluating and improving the success of various conservation interventions

One of the strengths of conservation physiology is its ability to provide objective scientific information to permit evaluation of the extent to which various conservation and management activities are successful. For example, restoration of degraded habitats is a common conservation action that is presumed to have benefits at a variety of biological levels. Traditional measures of community structure to assess success can be slow to respond to changes and often take longer than the period for which monitoring is set to occur (if any monitoring at all; Adams and Ham, 2011). Physiological tools can be used to understand whether there are individual-level benefits associated with restoration (e.g. reduced stress, improved growth, or nutritional condition) on a shorter time frame (Cooke and Suski, 2008). For example, Szota *et al.* (2011) contrasted the response of two eucalypts to seasonal drought at restored sites and determined that although the plants were in the same functional group, they responded differently to resource limitation. Success of restoration plans can be improved by using physiological knowledge about environmental thresholds of different species to identify the types of species or which populations (usually plants) might be likely to succeed, particularly in highly degraded sites (e.g. Pywell *et al.*, 2003; Vance *et al.*, 2003). Translocation of animals is another conservation strategy that has benefited from physiological knowledge. Research has revealed the best practices for relocating wildlife (e.g. Dickens *et al.*, 2010) and plants (e.g. Godefroid *et al.*, 2011), while minimizing stress and maximizing survival. Conversely, a recent ecosystem-level decision by the salmon-farming industry in British Columbia, Canada voluntarily to relocate salmon net pens away from the migration path of juvenile Pacific salmon during the migration window was based in part on physiological studies of swimming and osmoregulatory performance of pink salmon that characterized the critical role of fish size in tolerating parasitic sea lice (Brauner *et al.*, 2012).

## Conclusion

To date, various definitions of conservation physiology have existed, but all possessed limitations. In particular, some were not sufficiently inclusive in recognizing the role and full potential of conservation physiology. Here, we have refined the definition of conservation physiology by reflecting on past definitions and identifying the key requirements of the definition. As a result, the definition we have generated is, in many ways, an integration of previous definitions. Moreover, the definition was generated by a diverse authorship team covering plants and animals (from insects to vertebrates), as well as different topical expertise. To reiterate, conservation physiology is an integrative scientific discipline applying physiological concepts, tools, and knowledge to characterizing biological diversity and its ecological implications; understanding and predicting how organisms, populations, and ecosystems respond to environmental change and stressors; and solving conservation problems across the broad range of taxa, including microbes, plants, and animals. Physiology is considered in the broadest possible terms to include functional and mechanistic responses at all scales, and conservation includes the development and refinement of strategies to rebuild populations, restore ecosystems, inform conservation policy, generate decision-support tools, and manage natural resources.

We are confident that this new definition will be embraced by the broader scientific community. However, when dealing with biodiversity crises, and in the face of immense management and policy challenges, we recognize that action is more important than the definition (Robinson, 2006). We hope, therefore, that the new definition and journal will galvanize further action. Conservation physiology is certainly needed, and it has great potential to be effective by broadly contributing to conservation science in many ways (Cooke and O'Connor, 2010; Box 1). In addition, given that physiology embraces a variety of topical sub-disciplines and types of expertise (Table 2), there are many ways in which physiology can contribute. We cannot possibly identify all of the ways in which conservation physiology is needed, but the examples that we provide here represent ways in which tangible and rapid progress can be made (see Soulé and Orians, 2001). Indeed, it is our view that the creativity of those working on conservation physiology will identify new ways in which it can become even more relevant to conservation practitioners. Moreover, because of the ability of conservation physiology to generate cause-and-effect relationships, we anticipate a rapid expansion of its use in support of evidence-based conservation (Sutherland *et al.*, 2004). Indeed, evidence-based conservation is likely to run a parallel course to that taken by medical science, where mechanistic experimentation is currently the primary way in which the evidence base is established (Pullin and Knight, 2001).

For conservation science to reach its full potential will require true integration (Beissinger, 1990; Balmford and Cowling, 2006; Noss, 2006), which means that physiologists will need to collaborate with scientists with other expertise (i.e. integration; Fazey *et al.*, 2005), as well as directly with conservation practitioners (Lawler *et al.*, 2006; Cooke and O'Connor, 2010). The success of conservation science requires building on strengths and foundations of existing sub-disciplines, both basic and applied (Noss, 2006); physiology certainly provides such a foundation.
